# Promising Physical, Physicochemical, and Biochemical Background Contained in Peels of Prickly Pear Fruit Growing under Hard Ecological Conditions in the Mediterranean Countries

**DOI:** 10.1155/2019/9873146

**Published:** 2019-12-26

**Authors:** Mohammed Bourhia, Hamza Elmahdaoui, Riaz Ullah, Ahmed Bari, Laila Benbacer

**Affiliations:** ^1^Laboratory of Chemistry, Biochemistry, Nutrition, and Environment, Faculty of Medicine and Pharmacy, University Hassan II, Casablanca, Morocco; ^2^Laboratory of Food Technology and Quality, Regional Center for Agricultural Research in Marrakesh, National Institute for Agricultural Research, INRA, Marrakesh, Morocco; ^3^Medicinal Aromatic and Poisonous Plants Research Center, College of Pharmacy, King Saud University, P.O. Box 2457, Riyadh 11451, Saudi Arabia; ^4^Central Laboratory, College of Phamacy, King Saud University, P.O. Box 2457, Riyadh 11451, Saudi Arabia; ^5^Research Unit and Medical Biology, National Center for Nuclear Energy, Science and Technology, CNESTEN, Rabat 10001, Morocco

## Abstract

**Background:**

Prickly pear (*Opuntia* spp.), called Barbary fig, is a cultivated species springing from family *Cactaceae*. It is native to Mexico and has been naturalized in other continents, especially the Mediterranean countries (North Africa). The aim of the study was to investigate the physical, physicochemical, and biochemical criteria of peels of three Moroccan prickly pear varieties (Aakria, Derbana, and Mles) growing in the Rhamna regions (dry area).

**Material and Methods:**

Both physicochemical characteristics (humidity, water activity, Brix, ash content, pH, and total titratable acidity) and biochemical characteristics (total carotenoid content, betalain content, total polyphenolic content, and ascorbic acid content) were were studied according to previously reported methods.

**Results:**

Regarding the physiochemical criteria, the moisture of the fresh peels of studied varieties ranged from 81.59 ± 0.02 to 83.47 ± 0.02%. The water activity (aw) ranged from 0.862 ± 0.001 to 0.872 ± 0.001. The values of Brix varied from 14.69 ± 0.05° Bx to 15.80 ± 0.03° Bx. pH values varied from 5.13 ± 0.01 to 5.32. The total titratable acidity values ranged from 0.130 ± 0.008 to 0.196 ± 0.014 g of citric acid/100 g of FM (fresh matter). The ash content values ranged from 8.92 ± 0.10 to 11.04 ± 0.06 g/100 g of FM. Regarding the biochemical criteria, the total carotenoid content ranged from 2.29 ± 0.01 to 2.87 ± 0.01 *μ*g/g of FM. The total betalain content ranged from 6213.46 ± 58.86 to 8487.19 ± 51.71 *μ*g/100 g of FM. The total polyphenolic content varied from 160 ± 3.55 to 243.79 ± 5.55 mg GA E/100 g of FM. The ascorbic content ranged from 58.21 ± 0.24 to 74.72 ± 0.17 mg/100 g of FM.

**Conclusion:**

The findings of physicochemical and biochemical criteria of the investigated varieties growing in Moroccan drylands showed promising results in terms of studied parameters.

## 1. Introduction

Morocco is one of the several countries that have been subjected for many years to severe drought with adverse consequences on agriculture and general economic activities of the country. To face this environmental hazard, the targeted countries have obliged to develop agriculture more adapted and less influenced by climatic burdens [1].

Drought has become one of the biggest challenges that affect negatively sustainable crop production. Climate change such as desertification is the major challenge faced by Moroccan zone where the rural landscape is most heavily affected. Therefore, the crops which can resist to such hard conditions like, drought, high temperatures, and low soils need more emphasis.

Prickly pear crops are receiving increasing interest throughout the globe due to their unique criteria, which provide resilience to the hard ecological conditions. Prickly pear can grow on land where many other crops are unable to grow; it can be used to repair degraded land. It has the ability to adapt to stress even at low soil potential. It is the only plant that can flourish when others fail. Thus, it is an environment friendly plant which can resist long periods of dryness [2, 3].

The synthesis of secondary metabolites by the plants is positively influenced when plants grow under climate stress with hard environmental conditions during varying environments. The increase of secondary metabolites diversity is frequently conducted by environmental components that comprise local geo-climatic. Furthermore, seasonal changes and external conditions like temperature, light, humidity, and developmental processes are intensively involved in secondary metabolites diversity [4, 5].

Nowadays, the cultivation of prickly pear is intensively practiced in Morocco. Numerous efforts have been carried out by the state under the Green Morocco Plan to make Barbary fig an industrial crop for both animal and human feeding. In Morocco, the total area planted with prickly pear has increased from one year to the next. The extension of the cultivated area resulted in an increase in fruit production. The prickly pear crops and their affiliated services sectors can support large numbers of workers, provide them with sustainable jobs, and increase the prosperity of their neighbours and communities. An efficient agro-industry enhances economic stability for rural people, increases food safety, and helps achieve economic development [6].

The current research work was conducted to evaluate three varieties of Barbary fig growing in Moroccan drylands. The current study was deeply assessing the physical, physicochemical, and biochemical characteristics of three Moroccan perkily pear verities growing in the Rhamna regions under hard ecological conditions.

## 2. Material and Methods

### 2.1. Study Area

**Figure 1:**
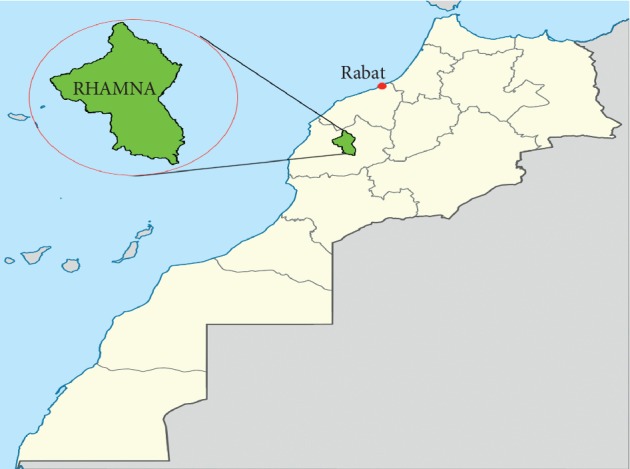
Geographical location of the study area (Rhamna region, Morocco).

The plant material was harvested from the rural areas of Skhour Rhamna. This sampling area is located in the region of Marrakech-Safi, Morocco. It is located 99 km north of Marrakech city (32°29′N: 7°55′W) (Figure 1).

### 2.2. Climatic and Soil Conditions

**Figure 2:**
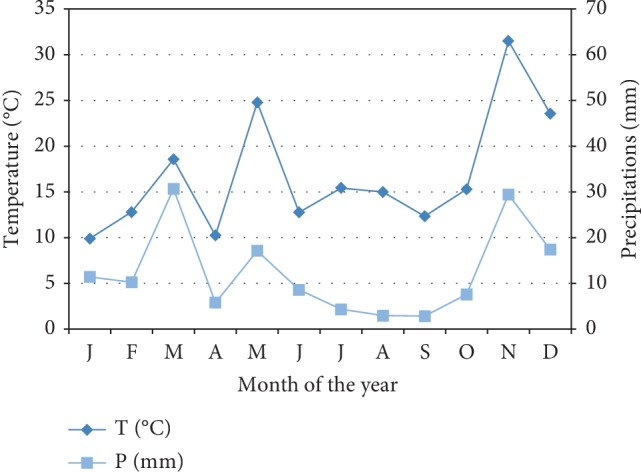
Gaussen and Bagnouls diagram of Rhamna region (2015-2016).

A low rainfall characterizes the study area (Skhour Rhamna region) over the year (150 to 350 mm). The number of dry months ranges from 8 to 11. The climax vegetation in the study area is composed of species well adapted to lack of water and less heterogeneous. Diagram of Gaussen and Bagnouls applied in Rhamna region shows a dry study area over the year (Figure 2).

### 2.3. Plant Material

**Figure 3:**
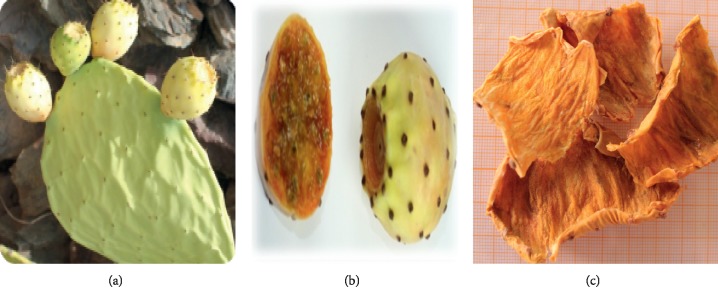
Variety of Mles (*Opuntia ficus-indica*). (a) Plant of *Opuntia ficus-indica* (Mles). (b) Fruit of *Opuntia ficus-indica* (Mles). (c) Peels of *Opuntia ficus-indica* (Mles).

**Figure 4:**
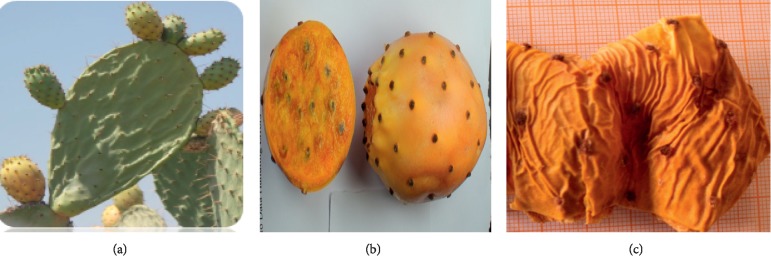
Variety of Derbana (*Opuntia megacantha*). (a) Plant of *Opuntia megacantha* (Derbana). (b) Fruit of *Opuntia megacantha* (Derbana ). (c) Peels of *Opuntia megacantha* (Derbana).

**Figure 5:**
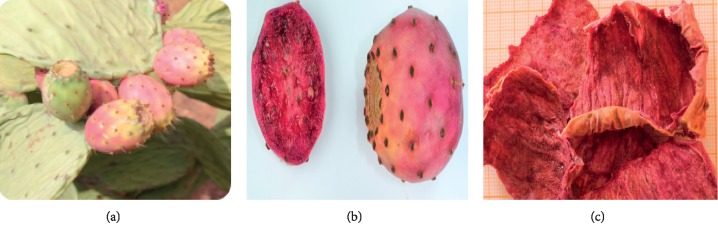
Variety of Aakria (*Opuntia ficus-indica*). (a) Plant of *Opuntia ficus-indica* (Aakria). (b) Fruit of *Opuntia ficus-indica* (Aakria). (c) Peels of *Opuntia ficus-indica* (Aakria).

The whole plants of three chosen varieties belonging to *Opuntia* spp., locally called Aakria, Derbana, and Mles (Figures 3–5), were collected at the maturity from the study area during the two crop years 2015-2016. The plant was identified by a botanist. The fruits of the studied plant were peeled. The obtained peels were crushed and saved at −20°C in order to avoid a probable alteration.

### 2.4. Physicochemical Analyses of Prickly Pear Peels

#### 2.4.1. Determination of Humidity

The moisture is frequently determined by drying the test portion until stabilization of weight [7]. 2 g of fresh peels was used for humidity determination.

Moisture percentage was determined according to the following formula:

(1) $$\begin{array}{c} \text{humidity}\%=\left(\frac{\text{Iw}-\text{Fw}}{\text{Iw}}\right)*100, \end{array}$$

where Iw is the test portion weight before heating (g) and Fw is the test portion weight after heating (g).

#### 2.4.2. Determination of Water Activity

The water activity (aw) currently describes the availability of “free” water in a product [8]. 5 g of fresh peels was measured at 25°C using an aw-meter apparatus. The result of water activity was recorded after 2 hours with respect to the required time to establish equilibrium.

#### 2.4.3. Determination of pH

The current pH was determined at 25°C by a pH meter after necessary calibration with buffer solution twice, pH 4 and pH 7. The current pH was determined directly in ground peels.

#### 2.4.4. Determination of Total Titratable Acidity

The total titratable acidity of fresh peels was measured as follows: an extract obtained from 25 g of test portion was supplemented with distilled water and heated for 30 minutes. After filtration, the filtrate was titrated again with a solution of NaOH (0.1 N) to pH = 8.1. The results were expressed in g of citric acid per 100 g of FM:

(2) $$\begin{array}{c} \text{total titratable acidity}\left(\frac{\text{g c. a}}{100\;g}\right)=\frac{\left(\text{VNaOH added}*\text{NNaOH}*0.\;07\right)/\text{Vs}}{\;100}*\text{Sw,} \end{array}$$

where VNaOH is the volume of NaOH (in ml), NNaOH is the normality of NaOH, Vs denotes the sample volume titrated (in ml), c.a denotes citric acid, 0.07 is the citric acid coefficient, and Sw denotes the sample weight.

#### 2.4.5. Brix Determination

The Brix scale is currently used to assess the soluble dry matter percentage in a product. 15 g of fresh peels was centrifuged at 12,000 rpm for 31 min. The obtained supernatant was used for the determination of Brix after the filtration procedure. Regarding dried peels analysis, the test portion was 1 g solubilized in 10 ml of distilled water at a ratio of 1 : 10 (powder of peels: distilled water). The findings were expressed in degree Brix (°Bx).

#### 2.4.6. Ash Content Determination

2 g of fresh peels was placed for incineration at 500°C for 7 hours. The ash content was calculated as a percentage relative to the dry matter (DM) with respect to the following formula:

(3) $$\begin{array}{c} \text{ashes in }\text{g}/100\text{ g}\text{ DM}=\left[\frac{\left(\text{Iw}-\text{Fw}\right)*100}{100-H}\right]*100, \end{array}$$

where Iw is the weight of the test sample before incineration (g), Fw is the weight of the test sample after incineration (g), and H denotes humidity (%).

### 2.5. Biochemical Analysis of Peels of Prickly Pear

#### 2.5.1. Total Carotenoid Content Determination

10 g of fresh peels of prickly pear was subjected to extraction (hexane/acetone/ethanol (50 : 25 : 25, v/v)). The obtained mixture was centrifuged again at 6500 rpm at 6°C for 6 min. The hexane fraction possessing pigments was recovered for measuring the total carotenoid. The total carotenoid content was measured by reading the absorbance at 450 nm. The findings were expressed in *μ*g equivalent *β*-carotene using the absorbance coefficient of 2500:

(4) $$\begin{array}{c} \text{total carotenoids }\left(\mu \text{g eq}.\beta ‐\text{carotene}/\text{g}\right)=\frac{A450*V*106}{2500*100*\text{Sw}}, \end{array}$$

where total carotenoids (*μ*g eq. *β*-carotene/g) = A450*∗V∗*106/2500*∗*100*∗*Sw, A450: absorbance at 450 nm, *V*: analyzed volume of the sample (1 ml), and SW: sample weight (g).

#### 2.5.2. Determination of Betalain Content

Assessment of betalain content was carried out according to the method reported in earlier literature [9]. 0.5 g of peels was extracted with 10 ml of methanol 80 % (v/v). The obtained extract was centrifuged at 4000 rpm for 21 minutes; the covered supernatant contained betalains.

Betalain content was measured according to the following equation:

(5) $$\begin{array}{c} \text{betalain content }\left(\mu \text{g}/100\text{ g}\right)=\left(\frac{\left(A*\text{Df}*\text{Mw}*1000/\xi *\text{Wt}\right)}{\text{Sw}*100}\right), \end{array}$$

where A is the absorbance, Df is the dilution factor, Wt is the width of the tank (1 cm), *ξ* is the molar extinction coefficient (L/mol *∗* cm), Mw denotes molecular weight (g/mol), *λ* denotes wavelength (nm), and Sw represents the sample weight.

#### 2.5.3. Determination of Total Polyphenol and Ascorbic Acid Content

*(1) Extraction*. 0.5 g of fresh peels was extracted with 10 ml of acetone (70%, 80%). The obtained extract was centrifuged at 5000 rpm for 31 min and then filtered. The total polyphenol and ascorbic acid contents were determined in the obtained extract.

#### 2.5.4. Determination of Total Polyphenols

The total polyphenolic content was measured with respect to the Folin–Ciocalteu method described in earlier published data with slight modifications [10]. The current assay used for determining the total polyphenols was conducted in triplicate, and the results were expressed in mg (GAE)/100 g of FM.

#### 2.5.5. Ascorbic Acid Content

A small sample of the current extract was mixed with 3.5 ml of distilled water. 2 ml of the present mixture was put on an OASIS cartridge previously covered with 3 ml of MeOH +2 *∗* 3 ml of distilled water. 0.5 ml of the current solution was dosed according to Folin–Ciocalteu method instructions. The findings were expressed in mg/100 g of FM.

### 2.6. Statistical Analysis

Quantitative data were analyzed using one-way ANOVA to compare the means for different studied varieties in the current work (Aakria, Derbana, and Mles). The Student–Newman–Keuls method was used to search the homogeneous groups of means. Data of the present work were presented as means ± standard deviation.

## 3. Results and Discussion

### 3.1. Physicochemical Characterization of Fruit Peels of Prickly Pear

Table 1 summarizes the physicochemical criteria of prickly pear fresh peels of the three studied varieties (Aakria, Derbana, and Mles) harvested in 2016 from the region of Rhamna. The current criteria were moisture, water activity, Brix, pH, total titratable acidity, and ash content.

#### 3.1.1. Humidity

The average of moisture content of fresh peels of Derbana and Aakria varieties was 81.59 ± 0.02 and 83.47 ± 0.02%, respectively. A high significant difference was found between the three studied varieties (*p* < 0.001) (Table 1). These results were in accordance with earlier data, in which it was reported that the value of moisture content of *Opuntia ficus-indica* fresh peels was 80.17 ± 0.93% [11].

The current levels of moisture content make these fresh peels highly perishable. Therefore, it is necessary to preserve them in order to avoid potential microbial deterioration. The difference in humidity level between the fruit peels of the three varieties could induce a significant difference in required energy and time for possible drying for better conservation.

#### 3.1.2. Water Activity

The values of water activity (aw) of fresh peels (0.862 ± 0.001 to 0.872 ± 0.001) were matched with moisture values. Both criteria of water activity and moisture are reflecting poor conservation ability due to microbial development at these intervals of water activity [12]. In regards to water activity, variance analysis showed that there is no significant difference between the two varieties of Derbana and Mles (Table 1).

The values of water activity reported in the current work were lower than those reported in earlier found data (aw = 0.895) regarding prickly peels of pear [13].

#### 3.1.3. Brix

Analysis of variance showed a significant difference between the fresh peels of prickly pear varieties (*p* < 0.001). The highest value of Brix was attributed to the variety of Derbana (15.80 ± 0.03° Bx) while the lowest was attributed to the variety of Aakria (14.69 ± 0.05° Bx) (Table 1). Similar results were reported for the fresh peels of *Opuntia ficus-indica* (15.00 ± 0.50° Bx) [14]. Due to its richness in soluble sugars, the peels of prickly pear are recommended for animal feeding [15].

#### 3.1.4. pH

The pH of the fresh peels was weakly acidic. A significant difference was reported between the three varieties (*p* < 0.001). The fruit peels of Aakria variety was the most acidic (pH = 5.13 ± 0.01) compared to the other varieties such as Derbana and Mles with pH values of 5.32 and 5.21, respectively (Table 1).

The current pH values were higher than those found previously in Algerian varieties of *Opuntia ficus-indica* (4.00 ± 0.01) [9]. This difference could be explained by the difference in the maturity stage of fruits, climatic and edaphic conditions, or varietal effect. The pH of fresh peels of the current *Opuntia* spp. was very close to that of orange peels (5.67 ± 0.01) [16].

#### 3.1.5. Total Titratable Acidity

Analysis of variance of total titratable acidity showed a significant difference between the fresh peels of Aakria variety and the other studied varieties (Derbana and Mles) (*p* < 0.001) (Table 1).

The obtained values of total titratable acidity ranged from 0.130 ± 0.008 to 0.196 ± 0.014 g of citric acid/100 g of FM. These results were in accordance with those reported in earlier published data regarding the total titratable acidity of fresh peels of *Opuntia ficus-indica* (0.12 ± 0.01 g citric acid/100 g FM) [11]. Due to their low acidity and their high levels of soluble sugars, the presently studied peels could be considered as a natural source for livestock feed.

#### 3.1.6. Ash Content

The statistical analysis showed a significant difference between the fresh peels of studied varieties (*p* < 0.05).

The ash content of fresh peels ranged from 8.92 ± 0.10 to 11.04 ± 0.06 g/100 g of FM. Aakria variety possesses higher mineral content compared to Derbana and Mles varieties (Table 1). The ash content of prickly pear peels obtained in the present study was in agreement with earlier reports showing that the ash content of Aakria variety was 11.5 g/100 g of FM [17]. The current findings were relatively lower than those found earlier (12.1 g/100 g of FM) [18].

### 3.2. Biochemical Characterization of the Fresh Peels of Prickly Pear Fruits

#### 3.2.1. Pigment Content

**Figure 6:**
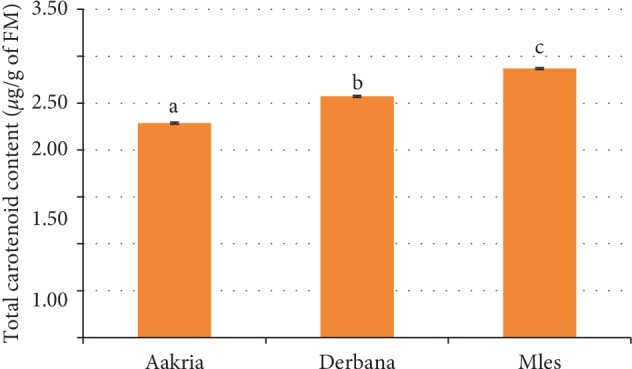
Total carotenoid content of fresh peels of prickly pear fruits (Aakria, Derbana, and Mles). Means with different letters differ significantly at *p* < 0.05.

*(1) Total Carotenoid Content*. Carotenoids are major determinants of organoleptic and nutritional qualities of fruits [19]. The fresh peels of studied varieties in the current work differ significantly in total carotenoid content (*p* < 0.001) (Figure 6). The results of total carotenoid content obtained in the current research ranged from 2.29 ± 0.01 to 2.87 ± 0.01 *μ*g/g of FM. These results were lower than those reported in earlier reports, 29.7 ± 0.2 *μ*g/g of FM [11]. Hence, this remarked difference could be related to the difference in the extraction method, chemicals, and the solvent used.

**Figure 7:**
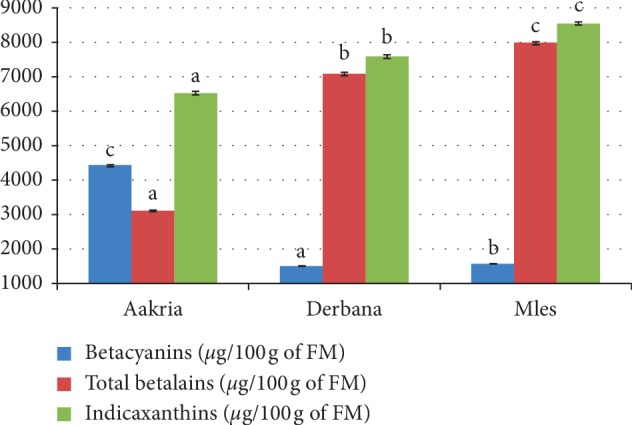
Content of betalains of the fresh peels of prickly pear fruits (Aakria, Derbana, and Mles). Means with the same letter did not present significant difference at *p* < 0.05.

*(2). Betalains*. The attractive colors of prickly pear are due to betalains including betacyanins (red-purple pigments) and betaxanthins (yellow-orange pigments) [20]. Analysis of variance showed a significant difference in total betalain content contained in the fresh peels of studied varieties (*p* < 0.001) (Figure 7).

Betacyanin content (red pigment) contained in the fresh peels of Aakria variety fruits was higher than that contained in Derbana and Mles varieties. However, indicaxanthin content (yellow pigment) contained in the fresh peels of Mles variety was higher than that contained in both Aakria and Derbana varieties. The total betalain content of Aakria and Mles was 6213.46 ± 58.86 and 8487.19 ± 51.71 *μ*g/100 g of FM, respectively. These values were in agreement with previous research carried out on *Opuntia sheeri* [21] in which it was reported that the value of betalain content was 8400 *μ*g/100 g of FM.

The important content of betalains revealed in peels of investigated varieties in this work could constitute a new way of prickly pear valorization further in the agribusiness and pharmaceutical sector. The betalains are considered as interesting natural dyes and powerful antioxidants [21]. Betalain pigments exhibit antioxidant, antiviral, and antimicrobial properties [22]. Betalains could be considered as cancer-preventive substances [23].

**Figure 8:**
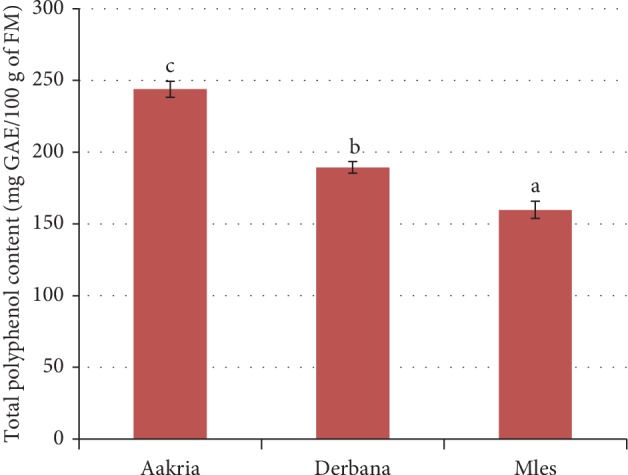
Phenolic content of fresh peels of prickly pear fruits (Aakria, Derbana, and Mles). Means with different letters present a significant difference at *p* < 0.05.

*(3). Content of Phenolic Compounds*. Regarding the content of phenolic compounds, a highly significant difference was noted between the fresh peels of all investigated varieties (*p* < 0.001). Indeed, the highest content was recorded in Aakria variety (243.79 ± 5.55 mg GAE/100 g of FM) (Figure 8).

The current results were in agreement with those reported in earlier data [24]; it was reported that the phenolic content of the fruit fresh peels of genus *Opuntia* was 226.2 mg GAE/100 g of FM. However, these results of phenolic content were relatively lower than those found in previous reports, which showed that the phenolic content of *Opuntia ficus-indica* fresh peels ranged from 250 to 300 mg EAG/100 g of FM [25].

The fresh peels of Aakria variety contained the highest rate of total polyphenols. Hence, this variety exhibits an important antioxidant potential compared to Derbana and Mles varieties. In addition, polyphenols could also play a protective role against chronic and cardiovascular diseases [26].

**Figure 9:**
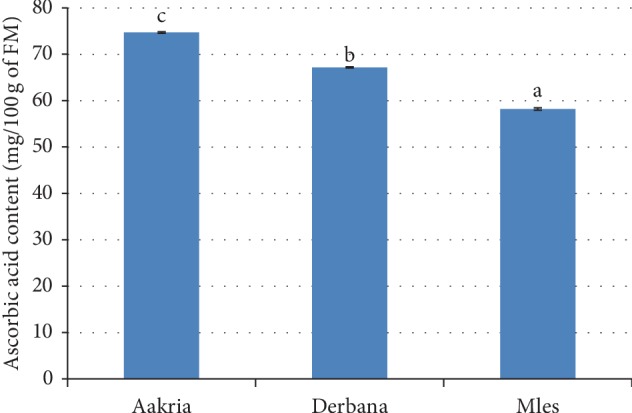
Ascorbic acid content of fresh peels of prickly pear fruits (Aakria, Derbana, and Mles). Means with different letters present a significant difference (Student–Newman–Keuls test at *p* < 0.05.

*(4). Ascorbic Acid Content*. Analysis of variance showed a significant difference between ascorbic acid content of studied varieties (*p* < 0.05) (Figure 9). The values of ascorbic content contained in the varieties of Mles and Aakria were 58.21 ± 0.24 and 74.72 ± 0.17 mg/100 g of FM, respectively. The current results revealed that the studied peels possess interesting bioactive compounds.

The biological properties of prickly pear peels attract researcher's attention for searching potentially interesting compounds [16]. These values were in agreement with those recorded in previous literature [27]. It was mentioned that the ascorbic acid content of *Opuntia ficus-indica* fresh peels was 59.82 mg/100 g FM. The bioactive compounds contained in prickly pear peels such as ascorbic acid have attracted scientists from different disciplines for screening their biological, pharmacological, and technological properties.

## 4. Conclusion

The present study gives big data on the physicochemical and biochemical criteria of fresh peels of three Moroccan prickly pear verities (Aakria, Derbana, and Mles). Both physicochemical and biochemical criteria of investigated varieties growing under stress climate in Moroccan drylands showed promising results in terms of studied parameters. Therefore, the present comparative study enables to underline that Darban variety was the best of the studied varieties in regard to its content of biochemical and physical parameters. All studied varieties may constitute a promising reservoir of natural compounds for potential use as natural food as well as in pharmaceutical fields.

## Figures and Tables

**Table 1:** Physicochemical criteria of prickly pear fresh peels of the studied varieties (Aakria, Derbana, and Mles).

Criteria	Fresh peels		
	Variety Aakria	Variety Derbana	Variety Mles
Humidity (%)	83.47 ± 0.02^c^	81.59 ± 0.02^a^	83.28 ± 0.01^b^
Water activity	0.872 ± 0.001^b^	0.862 ± 0.001^a^	0.864 ± 0.001^a^
Brix (°Bx)	14.69 ± 0.05^a^	15.80 ± 0.03^c^	15.49 ± 0.05^b^
pH	5.13 ± 0.01^a^	5.32 ± 0.01^c^	5.21 ± 0.01^b^
Total titratable acidity (g citric acid/100 g of FM)	0.196 ± 0.014^b^	0.130 ± 0.008^a^	0.146 ± 0.007^a^
Ash content (g/100 g of DM)	11.04 ± 0.06^c^	8.92 ± 0.10^a^	9.23 ± 0.05^b^

The findings are presented in means ± standard deviation. Values with the same letter in the same column have no significant difference at *p* < 0.05.

## Data Availability

All data are available in the following laboratories: Laboratory of Chemistry, Biochemistry, Nutrition, and Environment, Faculty of Medicine and Pharmacy, University Hassan II Casablanca, Morocco, Laboratory of Food Technology and Quality, Regional Center for Agricultural Research in Marrakesh, National Institute for Agricultural Research, INRA, Marrakesh, Morocco, and Research Unit and Medical Biology, National Center for Nuclear Energy, Science and Technology, CNESTEN, Rabat 10001, Morocco.
